# Identification and Fine-Mapping of *qPH15* for Plant Height in Sunflower (*Helianthus annuus* L.)

**DOI:** 10.3390/plants15101483

**Published:** 2026-05-13

**Authors:** Mingzhu Zhao, Dianxiu Song, Xiaohong Liu, Bing Yi, Yuxuan Cao, Jingang Liu, Dexing Wang, Liangshan Feng

**Affiliations:** 1 Institute of Crop Research, Liaoning Academy of Agricultural Sciences, Shenyang 110161, China; zhaomingzhu23@163.com (M.Z.);; 2 Liaoning Academy of Agricultural Sciences, Shenyang 110161, China

**Keywords:** sunflower, plant height, BSA-seq, fine mapping, *qPH15*, *HaNAC7*, haplotype analysis

## Abstract

Plant height is a key component of sunflower (*Helianthus annuus* L.) plant architecture. It strongly influences lodging resistance, mechanized harvestability, and yield stability. However, the genetic basis of plant height in sunflowers remains underexplored. This study aimed to develop an F_2_ population consisting of 715 individuals from a cross between the dwarf inbred line 150A and the tall inbred line PT326. Bulked segregant analysis coupled with whole-genome resequencing was employed to identify loci associated with plant height. Using three complementary analytical methods, a major quantitative trait locus, *qPH15*, was identified on chromosome 15. This locus was subsequently fine-mapped, using Kompetitive Allele Specific PCR (KASP) markers and recombinant screening in F_2_ and F_3_ populations, narrowing it to a 64.66-kb region containing three annotated genes. Among these, HanXRQr2_Chr15g0707451, which encodes an NAC transcription factor designated *HaNAC7*, was identified as the most promising candidate gene. Haplotype analysis of *HaNAC7* across 148 sunflower accessions revealed 4 polymorphic sites defining 6 haplotypes with substantial differences in plant height. The shortest haplotypes, Hap2 and Hap3, were associated with reduced plant height and were predominantly found in Asian germplasm. These findings suggest that *HaNAC7* is a strong candidate gene underlying *qPH15* and provide useful molecular markers and favorable allelic resources for improving sunflower plant architecture.

## 1. Introduction

Sunflower (*Helianthus annuus* L.) is one of the world’s most important oilseed crops and is also widely cultivated as a confectionery crop. In modern sunflower production systems, plant height is a key element of plant architecture, as it directly impacts the canopy structure, biomass distribution, lodging resistance, suitability for mechanized harvesting, and yield stability. As cropping systems continue to shift toward high-density planting and large-scale mechanization, sunflower breeding efforts are increasingly focused on optimizing plant height rather than simply reducing it. Therefore, analyzing the genetic basis of plant height is of great importance for agronomic improvement and biological research [1,2].

Plant height is a quantitative trait controlled by multiple loci and regulated by complex developmental and hormonal pathways. Previous studies in sunflower have identified sources of reduced height and several candidate genes, primarily associated with gibberellin biosynthesis or signaling pathways, such as *HaKAO1* and *HaDella1* [3,4]. Moreover, conventional linkage mapping and high-density marker analyses have revealed numerous quantitative trait loci (QTLs) associated with plant height across diverse sunflower populations [5,6]. Nevertheless, many of the loci reported in earlier studies are characterized by broad confidence intervals, strong dependence on genetic background, or inconsistent effects across environments. These limitations hinder the rapid identification of causal genes and the development of reliable molecular markers for breeding applications. Therefore, the discovery of novel major-effect loci and the elucidation of their underlying genetic mechanisms remain important tasks in sunflower plant architecture research.

The sunflower genome is large and repetitive, which has historically hindered high-resolution genetic dissection of complex traits. However, the availability of reference genome assemblies and the development of sequencing-based mapping strategies have greatly accelerated gene discovery in this species [1]. Among these approaches, bulked segregant analysis coupled with next-generation sequencing (BSA-seq/QTL-seq) has emerged as a powerful method for quickly identifying loci linked to quantitative traits by comparing allele-frequency differences between extreme phenotypic bulks. The integration of multiple analytical statistics, such as sliding-window ΔSNP-index (Index-slid), Gprime (G′), and Euclidean distance (ED), can further improve the robustness and reliability of mapping results [7,8,9,10].

Meanwhile, advances in mapping strategies have shifted attention toward transcription factor families that regulate plant architecture. Among these, the NAC family represents a large group of plant-specific transcription factors that participate in a wide range of developmental and stress-response processes, including meristem function, secondary cell wall formation, vascular differentiation, senescence, organ growth, and hormone-mediated developmental regulation [11,12,13,14]. In sunflowers, genome-wide analysis has indicated that the NAC family is both highly expanded and diverse. A comprehensive study identified 150 NAC genes in the sunflower genome, whereas a subsequent pangenome-based study further characterized 139 *HaNAC* genes and revealed significant haplotypic diversity within this gene family. These findings highlight the crucial role of NAC genes in sunflower trait variation and breeding [15,16]. Evidence from multiple plant species further supports that NAC transcription factors can influence plant height through diverse mechanisms, including the regulation of secondary cell wall biosynthesis, xylem and vessel formation, hormone signaling pathways, as well as cell division and expansion [17,18,19]. These findings suggest that NAC genes are strong candidates for architecture-related traits; however, their specific roles in regulating plant height in sunflowers remain largely unclear.

This study employed BSA-seq technology to identify major-effect QTLs regulating plant height in an F_2_ population derived from a cross between the dwarf inbred line 150A and the tall inbred line PT326. Based on the initial mapping results, the major-effect QTL *qPH15* was further fine-mapped, candidate genes within the refined interval were evaluated through expression analysis, and haplotype analysis was performed on the leading candidate gene *HaNAC7* across 148 sunflower accessions. By integrating rapid QTL mapping, fine mapping, gene expression profiling, and haplotype–phenotypic association analyses, this study aimed to identify a key gene influencing sunflower plant height. The findings provide valuable molecular resources and potential targets for ideotype breeding in sunflowers.

## 2. Materials and Methods

### 2.1. Plant Materials and Field Experiments

A population was developed by crossing the dwarf sunflower inbred line 150A with the tall inbred line PT326. The resulting F_1_ plants were self-pollinated to generate an F_2_ population. In June 2024, the F_2_ population, consisting of 715 individuals, was grown under field conditions at the Liaoning Academy of Agricultural Sciences, Shenyang, China (123.5° E, 41.8° N). In June 2025, an F_3_ population, comprising 2817 individuals, was developed from a heterozygous recombinant identified in the F_2_ generation cultivated at the same experimental site. The parental lines and F_1_ were planted in a randomized complete block design with three biological replicates. Each plot was planted in a two-row plot with a row length of 6 m. For the segregation of the F_2_ and F_3_ populations, 715 and 2817 individuals were also planted in the same field for phenotyping and genotyping-based mapping. Rows were arranged 50 cm apart, with plants spaced 60 cm apart within rows.

To assess allelic variation within the candidate gene region, a panel of 148 sunflower accessions was evaluated under field conditions in Shenyang during the 2024 and 2025 growing seasons. The experiment was conducted using a randomized block design with three replications. Each accession was grown in a two-row plot with a 6-m row length, using the same row and plant spacing described earlier. Border rows were planted surrounding the trial area, and field management followed standard local agronomic practices.

### 2.2. Phenotypic Evaluation and Construction of Extreme Bulks

The plant height of each F_2_ individual was measured at maturity. Based on the phenotypic distribution, 30 extremely dwarf and 30 extremely tall plants were selected from the 2 tails of the distribution to construct the dwarf and tall bulks, respectively. This sample size was chosen to balance phenotypic contrast, pool balance, and sequencing efficiency. The parental lines were also included for variant filtering and determination of parental allele origin. This extreme-phenotype pooling strategy was used to maximize allele-frequency differences between the two bulks, thereby enhancing the power and resolution of the BSA-seq analysis.

### 2.3. DNA Extraction, Library Construction, and Whole-Genome Resequencing

Young leaves were collected from individual plants, and genomic DNA was extracted using the Cetyltrimethylammonium Bromide (CTAB) method. Equal amounts of DNA from each selected plant were pooled to generate the dwarf and tall bulks, whereas parental DNA samples were processed individually. Paired-end sequencing libraries with an average insert size of approximately 350 bp were prepared and sequenced on an Illumina platform. Raw reads were subjected to adapter trimming, quality filtering, and standard quality control procedures to obtain clean reads for downstream analysis.

### 2.4. Read Alignment, Variant Calling, and Functional Annotation

Clean reads from the two bulks and the parental lines were aligned to the sunflower reference genome using Burrows–Wheeler Aligner (BWA) [20]. The resulting alignment files were converted, sorted, and indexed using Sequence Alignment/Map tools (SAMtools) [21]. Single Nucleotide Polymorphisms (SNPs) and Insertions and Deletions (InDels) were identified using the Genome Analysis Toolkit (GATK) pipeline and subsequently filtered based on sequencing depth, genotype quality, and missing rate [22].

Only biallelic SNPs and InDels were retained. Variants were required to meet the following thresholds: variant quality score (QUAL) ≥ 30, mapping quality (MQ) ≥ 30, and sufficient read support across all samples. Specifically, a minimum read depth of 10 was required for each parent, and a minimum read depth of 15 for each bulk. Sequencing quality was evaluated based on clean data yield, Q30 score, mapping rate, average sequencing depth, and genome coverage. Coverage uniformity was assessed by examining the breadth and consistency of reference genome coverage across the parental and bulked samples. Low-confidence loci and variants located in repetitive or ambiguous regions were removed. Functional annotation of filtered variants was performed using the SNP effect predictor (SnpEff) [23].

### 2.5. BSA-Seq Analysis and Identification of Candidate QTL Intervals

To improve mapping accuracy, filtered polymorphic loci were analyzed using three complementary BSA-seq statistics, including Index-slid, G′, and ED [7,8,9,10]. For the Index-slid analysis, allele frequencies at each locus were calculated in the two bulks, and the differences between bulks were smoothed using a sliding-window approach. Using the 0.999 quantile of the smoothed ΔSNP-index values as the significance threshold. The corresponding cutoff value was 0.723. For the G′ analysis, a tricube-smoothed G statistic was applied to identify the significantly associated genomic regions. Smoothed G′ values were calculated using a 1-Mb sliding window. The 0.999 quantile of the genome-wide smoothed G′ distribution was used as the threshold. The corresponding cutoff value was 13.371. For the ED analysis, ED values were computed from allele-frequency differences between the two bulks and then smoothed across adjacent loci. The raw ED values were first raised to the fourth power to enhance signal discrimination. The 99.9th percentile of the genome-wide smoothed ED^4^ distribution was used as the significance threshold, corresponding to a cutoff value of 1.755. For each statistic, only regions containing at least 10 variant sites above the threshold were retained as candidate intervals. Candidate genomic intervals were prioritized based on overlap among the three methods, with particular emphasis on regions supported by multiple statistics, following established BSA-seq and QTL-seq principles.

### 2.6. Development of KASP Markers and Fine Mapping of qPH15

To refine the major BSA-seq interval on chromosome 15, polymorphic SNPs within the *qPH15* region were converted into KASP markers. In the initial mapping step, nine KASP markers were developed within the preliminary BSA-seq interval and used to genotype the F_2_ population (Table S1). Recombinant individuals were identified by comparing marker genotypes with plant height phenotypes, enabling a substantial reduction in the *qPH15* interval.

For high-resolution fine mapping, a large F_3_ population derived from the heterozygous F_2_ recombinant F2-4 was screened using five additional KASP markers located within the refined region (Table S2). Recombinant F_3_ individuals were identified and classified based on their plant height phenotypes, and the final candidate interval was determined by integrating recombinant breakpoint positions with phenotype segregation.

### 2.7. RNA Isolation and Quantitative Real-Time Polymerase Chain Reaction Analysis

At the squaring stage, stem tissues were collected from the dwarf line 150A and the tall line PT326 for gene expression analysis. Total RNA was isolated from these stem samples, and cDNA was synthesized using the PrimeScriptRT Reverse Transcription Kit with genomic DNA removal (TaKaRa, Otsu, Japan). Quantitative real-time PCR (qRT-PCR) was performed on a LightCycler 480 II system (Vazyme Biotech, Nanjing, China), using actin as the internal control. Gene-specific primers were designed for the three annotated genes within the final *qPH15* interval. The primer sequences are listed in Table S3. Relative expression levels were calculated using the 2^−ΔΔCt^ method [24]. Three biological replicates and three technical replicates were included for each sample. Statistical significance was evaluated using Student’s *t*-test.

### 2.8. Haplotype Analysis of HaNAC7 in Sunflower Germplasm

To evaluate natural allelic variation at the candidate gene, four SNPs identified between the two parental lines (150A and PT326) within or near *HaNAC7* were used to design sequencing primers (Table S4). These four sites included two upstream SNPs, one nonsynonymous SNP in the coding region, and one downstream SNP. The allelic states of the four SNPs in the two parental lines, together with their corresponding haplotype assignments, are summarized in Table S8. Four representative polymorphic sites were selected and sequenced across the panel of 148 sunflower accessions. Sequence polymorphisms at these four sites were analyzed to define *HaNAC7* haplotypes, and the mean plant height for each haplotype was calculated using phenotypic data obtained in 2024 and 2025. Differences among haplotypes were evaluated by one-way ANOVA followed by multiple comparisons using Duncan’s test at *p* < 0.05. In addition, for single-marker analysis, accessions were grouped according to the allele carried at each SNP, and differences in plant height between allelic groups were tested using Student’s *t*-test.

## 3. Results

### 3.1. Phenotypic Distribution of Plant Height in the F_2_ Population

**Figure 1:**
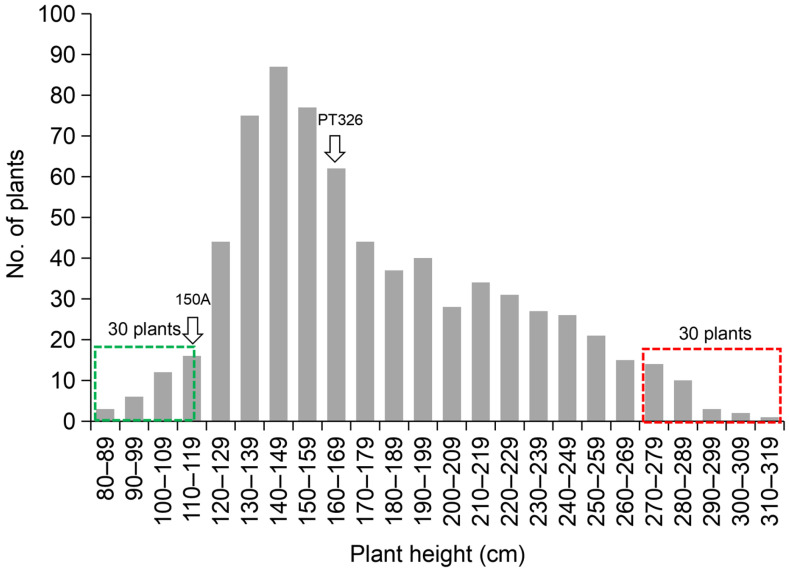
Phenotypic distribution of plant height in the F_2_ population derived from 150A × PT326. The histogram shows the frequency distribution of plant height (cm) among 715 F_2_ individuals. Dashed box indicate the selected extremes, including 30 dwarf plants (80–115 cm) and 30 tall plants (275–310 cm) used for BSA-seq bulks.

Phenotypic evaluation of the F_2_ segregating population, derived from the cross between the dwarf inbred line 150A and the tall inbred line PT326, showed clear quantitative variation in plant height (Figure 1). Plant height exhibited a continuous and approximately normal distribution, suggesting that it is a typical quantitative trait influenced by multiple genetic factors. A significant phenotypic peak was observed in the 140–149 cm range, indicating that, besides the polygenic background, one or more major-effect loci may substantially contribute to the observed variation in plant height within this population.

To enable quick identification of major loci associated with plant height, individuals from the extreme ends of the phenotypic distribution were selected for bulk construction. A total of 60 plants were selected, 30 being 80–115 cm (extremely dwarf) and another 30 being 275–310 cm (extremely tall), which were used to create the dwarf and tall bulks. These bulks, along with the two parental lines, were subjected to whole-genome resequencing and BSA-seq analysis.

### 3.2. Identification of Plant Height-Associated QTLs by BSA-Seq

To quickly map the major locus, we performed whole-genome resequencing on both parents and two extreme bulks, each consisting of 30 dwarf-type and 30 tall-type F_2_ individuals. High-quality sequencing data were generated for the parents and the two bulks (Table S5). The clean data per sample ranged from 74.05 Gb to 75.32 Gb, with Q30 values of 96.80–96.91%, average sequencing depths of 26.05×–28.34×, mapping rates of 99.68–99.81%, and genome coverage of 87.37–94.17%. These values indicate that the sequencing depth was sufficient, and the reference genome was broadly and fairly evenly covered across all samples.

Across the genome, a total of 8,048,809 SNPs and 1,112,658 InDels were identified between the two parental lines, of which 169,565 effective SNPs and 23,216 effective InDels were retained after filtering for downstream analysis (Table S6). Notably, chromosome 15 harbored the highest number of functional SNPs and InDels among all chromosomes, indicating the presence of genomic regions on this chromosome that may be strongly associated with the variation in plant height between the parents.

**Figure 2:**
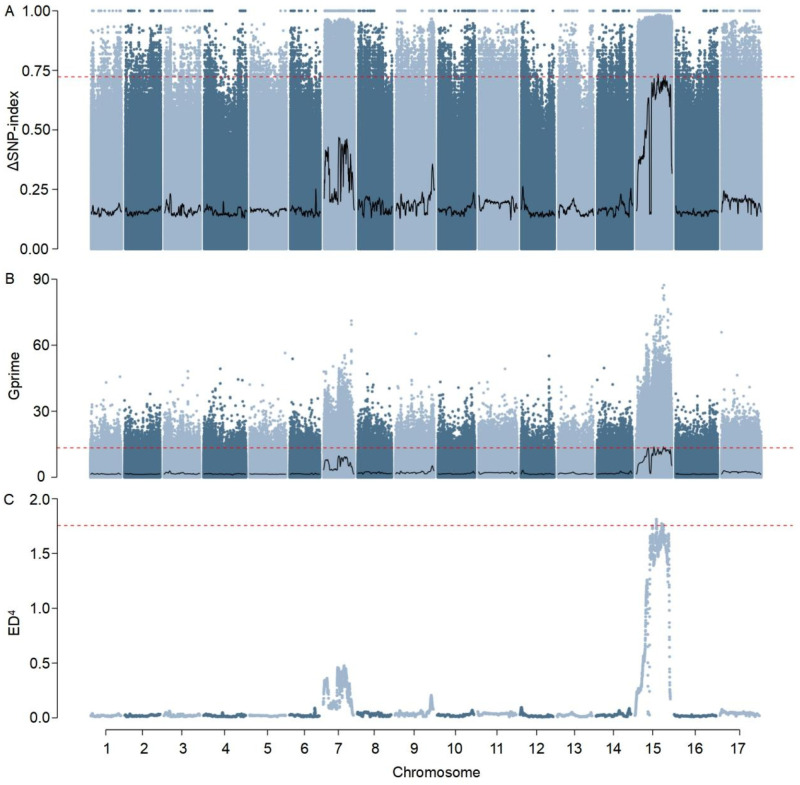
Identification of plant height-associated QTLs by BSA-seq. Three complementary statistics, including Index-slid (**A**), G′ value (**B**) and ED (**C**), were applied to detect allelic frequency differences between dwarf and tall bulks. The horizontal dashed lines represent the significance thresholds, with values of 0.723 for Index-slid, 13.371 for G′, and 1.755 for ED^4^. The red dashed lines indicate the significance thresholds for each statistic. Candidate regions were further required to contain at least 10 variants above the threshold.

To determine QTLs responsible for plant height, three distinct BSA analytical methods, including Index-slid, G′, and ED, were applied to the filtered genetic variants (Figure 2 and Table 1). All three methods detected a prominent association signal on chromosome 15, supporting the presence of plant height-related loci on this chromosome. No additional genomic regions passed the predefined significance threshold. Specifically, the ED method identified three candidate intervals, the G′ method identified two, and the Index-slid method identified two. Among these intervals, the region chr15:106,600,154–107,772,023 was consistently detected by both ED and Index-slid methods, showing complete overlap. The region encompassed 5543 SNPs, 1042 InDels, 108 functional SNPs, 11 functional InDels, and 42 annotated genes, highlighting that it is both highly polymorphic and gene-dense. Based on the principle of prioritizing intervals supported by multiple methods, this region was designated as the major-effect QTL for plant height, named *qPH15*.

### 3.3. Fine Mapping of qPH15 and Candidate Gene Identification

**Figure 3:**
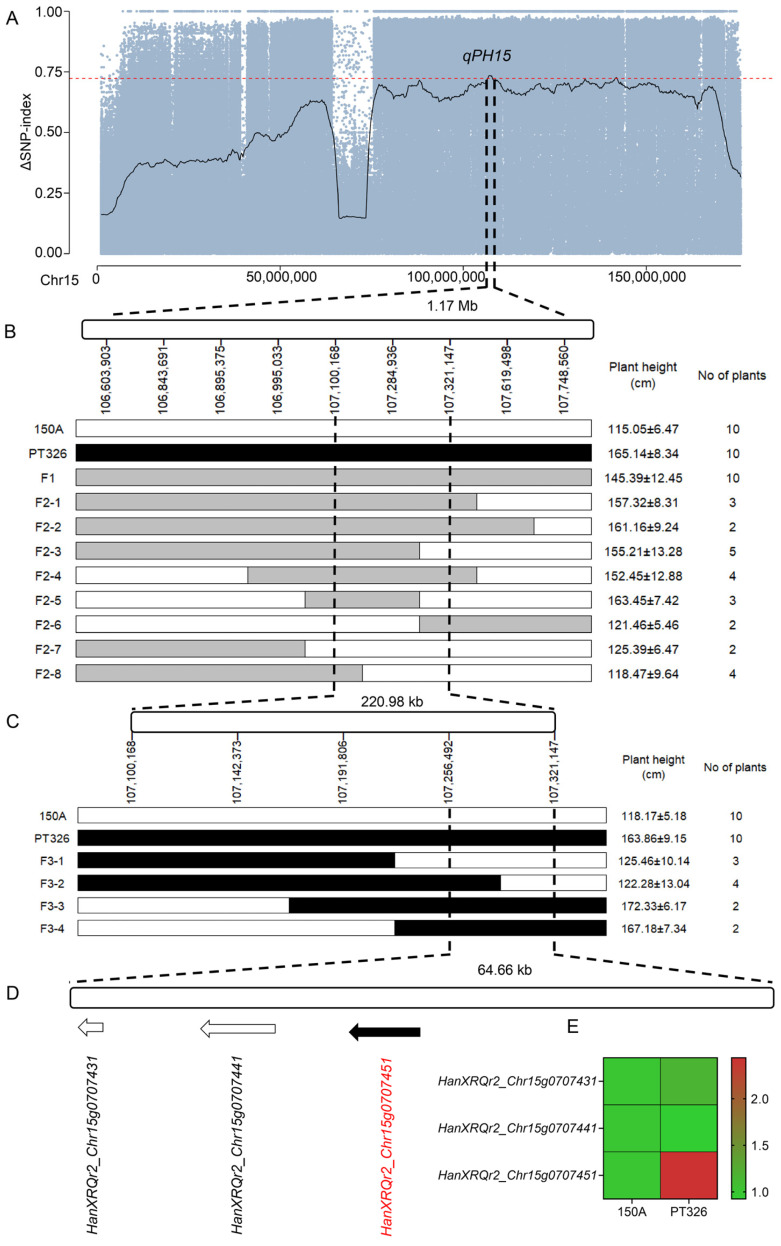
Fine mapping of *qPH15* and identification of *HaNAC7* as the candidate gene. (**A**) Initial 1.17 Mb interval on chromosome 15 delimited by BSA-seq. The red dashed lines indicate the significance thresholds for each statistic. (**B**) Recombination mapping in the F_2_ population using nine KASP markers narrowed the interval to 220.98 kb (chr15:107,100,168–107,321,147). Five recombinant individuals (F2-1 to F2-5) showed a tall phenotype, and three (F2-6 to F2-8) showed a dwarf phenotype. (**C**) High-resolution mapping in 2817 F_3_ individuals derived from heterozygous F2-4. Four additional recombinants (F3-1 to F3-4) delimited *qPH15* to a 64.66 kb region (chr15:107,256,492–107,321,147). (**D**) The final candidate interval contains three annotated genes. (**E**) qRT-PCR analysis of the three genes in stem tissues at the squaring stage.

The BSA-seq analysis initially delimited *qPH15* to a 1.17-Mb interval on chromosome 15 (Figure 3A). To further refine this locus, nine KASP markers were developed within the preliminary interval chr15:106,600,154–107,772,023. Genotyping of the F_2_ population identified eight recombinant individuals. Analysis of recombination breakpoints and associated phenotypes revealed that F2-1, F2-2, F2-3, F2-4, and F2-5 exhibited the tall phenotype, whereas F2-6, F2-7, and F2-8 showed the dwarf phenotype. These results narrowed the *qPH15* interval to chr15:107,100,168–107,321,147, spanning 220.98 kb (Figure 3B).

To further narrow the candidate interval, an F_3_ population derived from the heterozygous recombinant F2-4 was used for high-resolution mapping. A total of 2817 F_3_ individuals were genotyped using 5 KASP markers designed within the refined interval. Four additional recombinants were identified: F3-1 and F3-2 displayed the dwarf phenotype, whereas F3-3 and F3-4 exhibited the tall phenotype. Based on these genotypic and phenotypic relationships, *qPH15* was finally narrowed to a 64.66-kb interval, corresponding to chr15:107256492–107321147 (Figure 3C).

Within the final interval, only three annotated genes were present. Two genes, *HanXRQr2_Chr15g0707431* and *HanXRQr2_Chr15g0707441*, had no clear functional annotation, whereas *HanXRQr2_Chr15g0707451* encoded an NAC transcription factor, designated *HaNAC7* (Figure 3D). Expression analysis at the budding stage revealed no significant differences for *HanXRQr2_Chr15g0707431* and *HanXRQr2_Chr15g0707441* between the dwarf line 150A and the tall line PT326. In contrast, *HanXRQr2_Chr15g0707451* (*HaNAC7*) exhibited a markedly higher expression in the tall line PT326 compared with the dwarf line 150A (Figure 3E). These results strongly suggest that *HaNAC7* is the most likely candidate gene underlying the major plant height QTL *qPH15*.

### 3.4. Haplotype Analysis of the Candidate Gene HaNAC7

**Figure 4:**
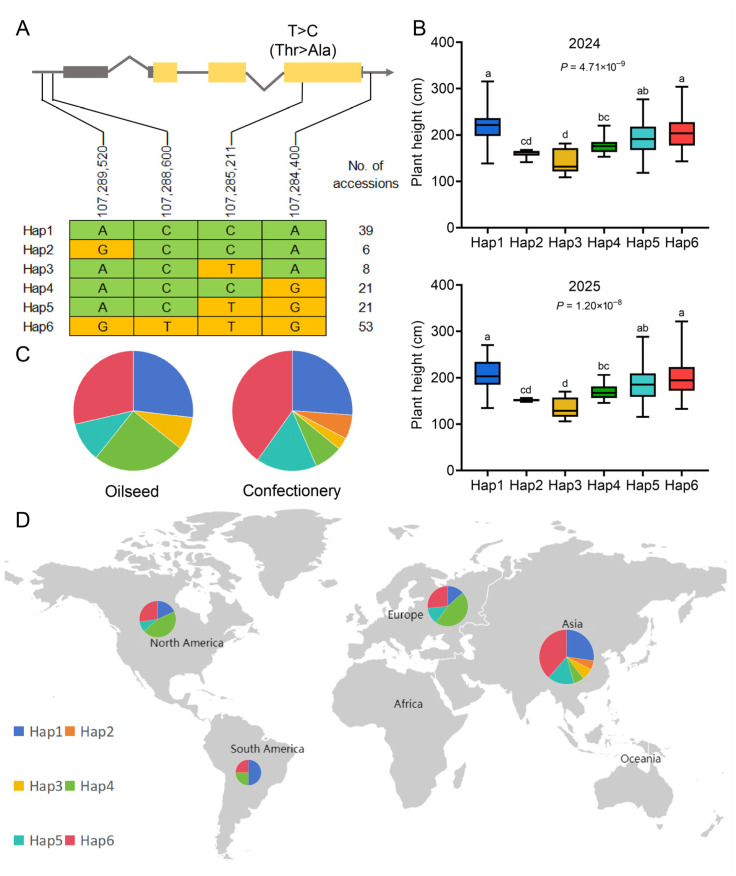
Haplotype analysis of *HaNAC7* in 148 sunflower accessions. (**A**) Schematic of the four polymorphic sites within or near *HaNAC7*. (**B**) Plant height distribution of the six haplotypes in 2024 and 2025. Different lowercase letters indicate significant differences among haplotypes based on Duncan’s multiple comparison test (*p* < 0.05). (**C**) Haplotype frequency comparison between oilseed and confection sunflower accessions. (**D**) Geographical distribution of haplotypes across continents.

To further assess the relationship between allelic variation in *HaNAC7* and plant height, haplotype analysis was conducted in 148 sunflower accessions (Table S7). Sequence analysis identified four SNPs within or near the *HaNAC7* locus: two SNPs located in the promoter region, one nonsynonymous SNP located in the exon resulting in an amino acid substitution (Thr to Ala), and one SNP located downstream of the gene (Figure 4A; Table S8). Based on these polymorphisms, the 148 accessions were grouped into 6 haplotypes, namely Hap1–Hap6, comprising 39, 6, 8, 21, 21, and 53 accessions, respectively. In addition, the parental genotypes at these four sites were clearly distinct, with 150A carrying the allelic combination corresponding to Hap6 and PT326 carrying the combination corresponding to Hap1 (Table S8).

Substantial phenotypic differences in plant height were observed among the six haplotypes (Figure 4B). Across 2 years of field evaluation, Hap3 exhibited the lowest plant height, with a height of 137.23 cm, followed by Hap2, which also conferred a relatively short phenotype, with a 2-year mean of 155.60 cm. In contrast, Hap1 was associated with the tallest plants, with average heights of 210.97 cm. Hap6 also conferred a relatively tall phenotype, with a mean height of 201.92 cm, followed by Hap5 (191.06 cm) and Hap4 (174.18 cm). These results indicated that allelic variation at the *HaNAC7* locus is strongly associated with plant height variation in sunflower. Importantly, the two haplotypes associated with reduced plant height (Hap2 and Hap3) may represent favorable allelic combinations for breeding shorter and potentially lodging-resistant ideotypes.

To further determine which of the four polymorphic sites was most strongly associated with plant height, each SNP was analyzed individually in the 148 accessions (Table S9). Among the four sites, chr15:107,288,600 showed a consistent and significant association with plant height in both years (*p* = 0.047 in 2024 and *p* = 0.035 in 2025), with accessions carrying the T allele being taller on average than those carrying the C allele. In contrast, the other three SNPs did not show significant differences between allelic groups in either year. These results suggest that chr15:107,288,600 may be the polymorphic site most strongly associated with plant height among the four tested markers.

The distribution of haplotypes also differed markedly between oilseed sunflower and confection sunflower accessions (Figure 4C). The dwarf-associated haplotype Hap2 was detected exclusively in confection sunflower (6.5%) and was absent from oilseed sunflower. Hap6 was the most abundant haplotype in confection sunflower (40.2%) and also common in oilseed sunflower (28.6%). By contrast, Hap4 was more frequent in oilseed sunflower (25.0%) than in confection sunflower (7.6%). Hap1 occurred at similar frequencies in both groups (26.8% in oilseed and 26.1% in confection), whereas Hap5 was more common in confection sunflower (16.3%) than in oilseed sunflower (10.7%). Hap3, associated with the shortest haplotype, was rare in both groups but slightly more common in oilseed sunflower (8.9%) than in confection sunflower (3.3%). These results suggest that allelic variation in *HaNAC7* has undergone differential selection during oilseed and confection sunflower breeding.

Geographical distribution analysis revealed a strong population structure for *HaNAC7* haplotypes (Figure 4D). The two dwarf-associated haplotypes, Hap2 and Hap3, were detected exclusively in Asia. In contrast, the tallest haplotype, Hap1, was most frequent in South America (50.00%), while Hap4 predominated in North America (45.45%) and Europe (46.67%) and was also common in South America (25.00%). Hap6 was widely distributed, with its highest representation in Asia (38.60%), reflecting its overall high frequency in the germplasm panel. Hap5 was also mainly distributed in Asia (15.79%) and rare or absent in other regions. These results indicate that *HaNAC7* haplotypes display clear geographic differentiation, with dwarf-associated haplotypes largely restricted to Asian sunflower germplasm.

## 4. Discussion

Plant height is a key component of sunflower architecture. In this study, BSA-seq combined with fine mapping narrowed the major-effect QTL *qPH15* to a 64.6-kb interval, and subsequent expression analysis combined with haplotype-based phenotypic associations supported *HaNAC7* as a strong candidate gene underlying this locus. As a NAC transcription factor, *HaNAC7* provides a biological link to the regulation of plant growth and architecture, consistent with the known roles of the NAC family functions in controlling developmental processes and structural traits. In addition, because plant height is a typical quantitative trait, the contribution of minor-effect loci or broader polygenic background cannot be excluded, even though chromosome 15 showed the strongest and most consistent signal in the present study. This point is also relevant when interpreting the relationship between parental phenotypes and haplotype means in the diversity panel because the phenotypic contrast between the two parents reflects the combined effects of *qPH15* and the broader genetic background.

NAC transcription factors constitute a large family of plant-specific regulators involved in a broad spectrum of developmental processes, including meristem maintenance, vascular differentiation, secondary cell wall synthesis, senescence, and responses to environmental stimuli [11,12]. Many NAC proteins play key roles in determining plant height, acting as transcriptional regulators during stem development. In *Arabidopsis*, *SND1*, *NST1*, and *NST3* serve as principal regulators of secondary wall formation in fibers and woody tissues, thereby enhancing stem strength and promoting vascular maturation [17,18,19]. Because during plant growth, stem elongation and mechanical support are closely coordinated, NAC genes occupy a key regulatory node that connects structural development with overall plant architecture.

Beyond their structural functions, NAC transcription factors also impact plant height by modulating hormonal pathways [19]. In rice, *OsNAC2* influences plant height through its involvement in gibberellin-related signaling [25], while *OsNAC103* suppresses plant height by regulating cell cycle progression and interacting with phytohormone networks [26]. These observations suggest that NAC genes integrate developmental and hormonal signals to control internode elongation and overall plant stature. Thus, the identification of *HaNAC7* in sunflower aligns with this growing body of evidence from other plant species, highlighting NAC transcription factors as important regulators of plant height–related traits.

Evidence from woody plants further reinforces the role of NAC genes in regulating plant height. In pear, *PbNAC71* controls dwarfism by repressing xylem and vessel development, suggesting that altered vascular differentiation is directly linked to reduced plant height [27]. In apple, overexpression of *MdNAC1* resulted in dwarf plants with shortened internodes and altered hormonal status [28]. These examples show that NAC genes can act as either positive or negative regulators of plant height, depending on species background, developmental context, and downstream targets. In the present study, the higher expression of *HaNAC7* in the tall parent indicates that this NAC gene likely functions as a positive regulator of stem growth in sunflower, potentially by enhancing vascular development, strengthening stem tissues, or synchronizing cell wall biosynthesis with elongation processes.

Given that *HaNAC7* encodes an NAC transcription factor, it is possible that it participates in regulatory processes affecting stem development and plant height. Previous studies in other species have suggested that NAC transcription factors can be involved in pathways related to stem development and growth regulation [29]. However, whether *HaNAC7* acts through similar mechanisms in sunflower remains unknown and was not directly tested in the present study. The *qPH15* locus in sunflowers appears to be distinct from previously identified height-reducing loci, such as *HaDella1* or *HaKAO1* [3,4], thereby broadening the current understanding of the genetic basis of plant height variations in sunflower.

Importantly, the current results supported the candidacy of *HaNAC7* at the locus level, but they do not yet resolve the underlying molecular mechanism by which this gene may influence plant height. *HaNAC7* haplotype analysis provides a valuable population–genetic perspective to this interpretation. Notably, the four polymorphic sites used to define these haplotypes were originally identified as sequence differences between the dwarf parent 150A and the tall parent PT326, and the two parents corresponded to Hap6 and Hap1, respectively. Interestingly, although the two parents differed significantly in plant height, the mean plant heights of Hap6 and Hap1 in the 148 accession panel were not significantly different from each other. This apparent discrepancy is biologically reasonable and does not weaken the candidacy of *HaNAC7*. The parental contrast reflects the net phenotypic effect of the *HaNAC7* region together with differences at many other loci across the genome, whereas the haplotype analysis estimates the average effect of the local *HaNAC7* haplotype across diverse genetic backgrounds.

Six haplotypes were identified, defined by four polymorphic sites located within or near the gene, and these haplotypes exhibited a clear association with plant height variations across the 148-accession panel. At the same time, not all haplotype pairs showed significant differences, indicating that the phenotypic effect captured by the four-site haplotype structure is informative but not sufficient to fully explain all observed variation in plant height. The mean height ranged from 137.23 cm for Hap3 to 210.97 cm for Hap1, demonstrating a substantial phenotypic effect attributable to allelic variation at this locus. Nevertheless, the present haplotype evidence does not by itself pinpoint the causal polymorphism, and the observed phenotypic differences may reflect direct functional effects, linkage disequilibrium, or a combination of multiple variants within the haplotype block. In particular, the lack of a significant difference between Hap1 and Hap6, despite the strong parental contrast between PT326 and 150A, further supported the view that variation outside the *HaNAC7* haplotype block or interactions with genetic background may substantially influence the final plant height phenotype.

The additional single-marker analysis provided further resolution of this pattern. Among the four polymorphic sites, the upstream SNP chr15:107,288,600 showed the most consistent association with plant height across both years, suggesting that regulatory variation may play an important role in the phenotypic effect of this locus. By contrast, although chr15:107,285,211 causes a nonsynonymous substitution, it did not show the strongest individual association in the present analysis. This result suggested that the observed height difference may not be explained solely by protein sequence change. However, this result should be interpreted as identifying the most informative polymorphic site within the tested haplotype block, rather than as fully accounting for the entire phenotypic divergence between the two parental lines.

From a breeding perspective, the SNP chr15:107,288,600, which showed the most stable association with plant height across two years, represents a promising candidate for the future development of a diagnostic marker for marker-assisted selection in sunflower. In particular, this site, or the most informative four-site haplotype combination, could be converted into a breeder-friendly assay such as a KASP marker. However, its practical predictive value still needs to be validated in broader breeding populations and genetic backgrounds before routine application, especially because the phenotypic effect of the *HaNAC7* haplotype appears to be influenced by background-dependent variation.

The distribution of *HaNAC7* haplotypes across market classes aligns with the known divergence between oilseed and confectionery sunflower. Confectionery sunflower breeding generally targets large, elongated seeds with lower oil content, easier dehulling, and a relatively tall plant type, with reported breeding targets around 175 cm in plant height, whereas oilseed breeding prioritizes oil yield and related agronomic traits [30,31]. Rather than assuming a universal target such as 175 cm, the optimal plant height for confection sunflower should be considered context-dependent and shaped by breeding objectives, production systems, and genetic background. This point is supported by our own haplotype data, in which Hap6, the most common haplotype in confection sunflower, had a mean plant height of 201.92 cm, indicating that currently prevalent confection germplasm does not conform to a single fixed height target. In this study, the presence of the short-stature Hap2 in confection sunflower and the enrichment of the intermediate Hap4 in oilseed sunflower suggest that allelic variation at *HaNAC7* may reflect differing architectural preferences between the two distinct market classes. However, the fact that Hap6, a relatively tall haplotype, was the most common in confection sunflower indicates that the selection at this locus was influenced by not only plant height but also broader ideotype considerations, including seed characteristics, standability, and adaptation to specific production systems. Therefore, the breeding value of shorter haplotypes such as Hap2 and Hap3 should be interpreted as providing useful allelic resources for adjusting plant stature, rather than as moving all confection sunflowers directly toward a predefined target such as 175 cm.

The geographical distribution of *HaNAC7* haplotypes provides additional evidence of regional differentiation at this locus. The two shortest haplotypes, Hap2 and Hap3, were detected exclusively in Asian germplasm, whereas Hap1 was the most frequent in South America, and Hap4 predominated in North America and Europe. This pattern is biologically plausible since confection sunflower has a strong production and consumption base in China as well as in other parts of Asia, such as Russia, Ukraine, Hungary, Israel, Spain, and Turkey [32]. The popularity of snack-seed sunflowers in China, Eastern Europe, and the Middle East further suggests that regional consumer preferences might have influenced breeding priorities for plant architecture and seed type [33]. Therefore, the confinement of Hap2 and Hap3 to Asia implies that reduced-height alleles of *HaNAC7* might have been favored in Asian breeding pools, particularly for confectionery sunflower.

The short-stature haplotypes, Hap2 and Hap3, might represent valuable allelic resources for enhancing compact plant architecture. However, their breeding value must be assessed within the context of the target germplasm, as their phenotypic effects may vary depending on interactions with other loci selected in oilseed or confectionery breeding pools. Moreover, because the number of accessions sampled from different regions was not fully balanced, the observed geographic patterns should be interpreted cautiously and validated in larger panels.

## 5. Conclusions

This study combined BSA-seq, fine mapping, expression analysis, and haplotype analysis to dissect the genetic basis of plant height in sunflower. A major-effect QTL, *qPH15*, was identified on chromosome 15 and progressively narrowed from a 1.17-Mb interval to a 64.66-kb candidate region. Within this interval, the NAC transcription factor gene *HaNAC7* emerged as a strong candidate underlying *qPH15*, based on its functional annotation and significant differential expression between the parental lines. Haplotype analysis further demonstrated that natural variation at *HaNAC7* is strongly associated with plant height variation across a panel of 148 accessions. Six haplotypes were identified, with the dwarf-associated Hap2 and Hap3 showing clear potential for breeding shorter plant ideotypes. The contrasting haplotype frequencies between oilseed and confection sunflower, together with their geographic differentiation, suggested that variation at or near *HaNAC7* may have contributed to the diversification of sunflower plant architecture during breeding and regional adaptation. Collectively, these results provided new insights into the genetic control of sunflower plant height and identified *HaNAC7* as a strong candidate gene for marker-assisted improvement of plant architecture.

## Figures and Tables

**Table 1:** Putative QTLs for plant height detected by three BSA association methods.

Method	QTL Region	Total SNPs	Effective SNPs	Total InDels	Effective InDels	Annotated Genes
Euclidean	chr15:133,000,320…133,124,196	733	1	53	1	1
Euclidean	chr15:141,601,090…141,794,118	539	0	30	0	2
Euclidean	chr15:106,600,154…107,772,023	5543	108	1042	11	42
Gprime	chr15:133,034,857…135,504,866	11,462	230	1354	26	54
Gprime	chr15:141,976,339…142,751,995	3127	45	320	11	9
Index-slid	chr15:106,600,154…107,772,023	5543	108	1042	11	42
Index-slid	chr15:141,410,274…141,794,118	1396	0	107	0	2

## Data Availability

The original contributions presented in the study are included in the article/Supplementary Materials; further inquiries can be directed to the corresponding author.

## Supplementary Materials

The following supporting information can be downloaded at: https://www.mdpi.com/article/10.3390/plants15101483/s1, Table S1. KASP markers developed for fine-mapping of qPH15 on chromosome 15 in F_2_ population. Table S2. KASP markers developed for fine-mapping of qPH15 on chromosome 15 in F_3_ population. Table S3. The primer sequences for quantitative real-time PCR analysis. Table S4. Primers used for sequencing the four polymorphic sites within or near HaNAC7 in the 148 sunflower accessions. Table S5. Summary of sequencing data quality for parental lines and extreme bulks. Table S6. Distribution of homozygous polymorphic SNPs and InDels between parents on each chromosome. Table S7. List of the 148 sunflower accessions used for haplotype analysis of HaNAC7. Table S8. Allelic states, functional annotations, and haplotype assignments of the four *HaNAC7* polymorphic sites in the parental lines 150A and PT326. Table S9. Single-marker association analysis of the four HaNAC7 polymorphic sites with plant height in 148 sunflower accessions in 2024 and 2025.
