# Supplementary material for: Identification and Fine-Mapping of qPH15 for Plant Height in Sunflower (Helianthus annuus L.)
Source: Plants (Basel). 2026 May 13;15(10):1483. doi: 10.3390/plants15101483 (PMC13210690; doi:10.3390/plants15101483)
Supplement: Supplementary file 1 [file plants-15-01483-s001.zip › plants-4276454-supplementary.pdf]

Table S1 KASP markers developed for fine-mapping of *qPH15* on chromosome 15 in F_2 population.

Chr	Position	Primer_Allele X	Primer_Allele Y	Primer_Common
chr15	106,603,903	GAAGGTGACCAAGTTCATGCTAATCACGTTCGCTAGCATCCACA	GAAGGTCGGAGTCAACGGATTAATCACGTTCGCTAGCATCCACG	TGGGGATTATGTCGAAGTGATAAAA
chr15	106,843,691	GAAGGTGACCAAGTTCATGCTGATGGAACAAAAAGGGGAGATGGG	GAAGGTCGGAGTCAACGGATTGATGGAACAAAAAGGGGAGATGGA	GCTACATGCACTCATCATCATATCC
chr15	106,895,375	GAAGGTGACCAAGTTCATGCTAGAAGCTGAGCGGCTTTTAAGATCA	GAAGGTCGGAGTCAACGGATTAGAAGCTGAGCGGCTTTTAAGATCG	GCCCACGTTCCTTTCCATAATTTCT
chr15	106,995,033	GAAGGTGACCAAGTTCATGCTCACGATCCTTTACGTCCTCAATCAC	GAAGGTCGGAGTCAACGGATTCACGATCCTTTACGTCCTCAATCAT	GTGATTGGAGCAGAGTCTCCATTG
chr15	107,100,168	GAAGGTGACCAAGTTCATGCTCTTGTGTGCCTCATTTCAACAACT	GAAGGTCGGAGTCAACGGATTCTTGTGTGCCTCATTTCAACAACA	GAGTTCAATGATCGGAACACAGAG
chr15	107,284,936	GAAGGTGACCAAGTTCATGCTCCAACTAAAGAAAATCATTGCACG	GAAGGTCGGAGTCAACGGATTCCAACTAAAGAAAATCATTGCACA	ATCATGTGGGCGTAATTGGGAATCT
chr15	107,321,147	GAAGGTGACCAAGTTCATGCTGACTGTCCGTTGGTGAACATCTTT	GAAGGTCGGAGTCAACGGATTGACTGTCCGTTGGTGAACATCTTG	CCGATACATAAGGCTTTGACACGAG
chr15	107,619,498	GAAGGTGACCAAGTTCATGCTAGATGTGAAACTAAAATACGTGGCA	GAAGGTCGGAGTCAACGGATTAGATGTGAAACTAAAATACGTGGCT	GGTTGGTTGATTCCTTATTGCCTTC
chr15	107,748,560	GAAGGTGACCAAGTTCATGCTCGTAGTAAGCCAACATGTCCCCAG	GAAGGTCGGAGTCAACGGATTCGTAGTAAGCCAACATGTCCCCAA	CCAGTTAAAGAAAATGGGTCGATCA

Table S2 KASP markers developed for fine-mapping of *qPH15* on chromosome 15 in F_3 population.

Chr	Position	Primer_Allele X	Primer_Allele Y	Primer_Common
chr15	107,100,168	GAAGGTGACCAAGTTCATGCTCTTGTGTGCCTCATTTCAACAACT	GAAGGTCGGAGTCAACGGATTCTTGTGTGCCTCATTTCAACAACA	GAGTTCAATGATCGGAACACAGAG
chr15	107,142,373	GAAGGTGACCAAGTTCATGCTCAGTTCAAATAACAGAGGTTGGTAA	GAAGGTCGGAGTCAACGGATTCAGTTCAAATAACAGAGGTTGGTAG	GTGAATGCACACTTGGCACA
chr15	107,191,806	GAAGGTGACCAAGTTCATGCTGCTCGGTGCAGTCTTTCTTAG	GAAGGTCGGAGTCAACGGATTGCTCGGTGCAGTCTTTCTTAC	GGGTTTGGGCGGAGGTAAAT
chr15	107,256,492	GAAGGTGACCAAGTTCATGCTGGGTGCTTAGAGGGAATGATAATT	GAAGGTCGGAGTCAACGGATTGGGTGCTTAGAGGGAATGATAATC	CCAGTTTTCGGTTGATCCAGG
chr15	107,321,147	GAAGGTGACCAAGTTCATGCTGACTGTCCGTTGGTGAACATCTTT	GAAGGTCGGAGTCAACGGATTGACTGTCCGTTGGTGAACATCTTG	CCGATACATAAGGCTTTGACACGAG

Table S3 The primer sequences for quantitative real-time PCR analysis.

ID	Forward (5'-3')	Reversed (5'-3')
HanXRQr2_Chr15g0707431	AACGAACAAAACCGTGGACTCAAGA	TCCTCTAATGCTTCAGCTTGATCAAAA
HanXRQr2_Chr15g0707441	TCTATTTTACTTCAACCATAGCTGAATT	AATTACTTGCTCAGGTGAAAGCGAATCC
HanXRQr2_Chr15g0707451	ACTTCATTATAGGTTTCTTTCTTCAT	AATCTGACTTTTGTCCATTCGGAGCACGA
Actin	GGAACAGGAATGGTGAAGGC	TCCATGTCATCCCAGTTGCT

Table S4 Primers used for sequencing the four polymorphic sites within or near HaNAC7 in the 148 sunflower accessions.

Chr	Position	Forward (5'-3')	Reversed (5'-3')
chr15	107,289,520	GATCAACCGACTAGGGAGCG	TTACCGGTGGCATTTTCCGA
chr15	107,288,600	ACCAATGGGAGACTGGCATG	TTGTAGCGACTACTCGGGGA
chr15	107,285,211	TCCTTCTTCAAGCACCCTCAC	AGGAAGAAGGGTGGGTGGTA
chr15	107,284,400	TAGTCAAGCCCGGCCAAAAA	AAGTGACGGATTGGCGAGTT

Table S5 Summary of sequencing data quality for parental lines and extreme bulks.

Sample ID	Raw Reads	Raw Bases （bp）	Raw GC （%）	Raw Q30 （%）	Clean Reads	Clean Bases （bp）	Clean GC （%）	Clean Q30 （%）	Mapped Ratio(%)	Proper Ratio(%)	Insert Size	Real Depth	Coverage(%) (>=1x)	Coverage(%) (>=4x)
150A	250,850,150	75,756,745,300	38.99	96.42	249,420,611	75,217,208,462	38.98	96.80	99.81	95.9	302	27.55	90.55	87.47
PT326	249,694,485	75,407,734,470	38.67	96.38	247,946,239	74,763,693,206	38.61	96.83	99.69	93.1	293	28.34	87.37	82.22
Dwarf type	247,184,823	74,649,816,546	38.85	96.36	245,543,128	74,047,644,850	38.83	96.80	99.68	94.25	304	26.05	94.16	91.13
Tall type	251,307,754	75,894,941,708	38.76	96.50	249,771,747	75,320,565,896	38.72	96.91	99.77	94.74	290	26.51	94.17	91.31

Table S6 Distribution of homozygous polymorphic SNPs and InDels between parents on each chromosome.

Chromosome	SNP Number	Effective SNP	InDel Number	Effective InDel
chr1	393,340	9,844	59,361	1,436
chr2	486,130	9,732	65,524	1,339
chr3	327,582	7,353	47,047	1,021
chr4	427,341	9,200	58,875	1,262
chr5	625,568	11,691	81,997	1,515
chr6	338,190	7,048	48,171	950
chr7	351,407	7,851	48,862	1,033
chr8	412,111	9,082	57,983	1,360
chr9	487,831	11,412	71,025	1,640
chr10	485,330	9,943	67,431	1,375
chr11	689,355	12,940	91,081	1,649
chr12	298,275	6,694	44,750	966
chr13	404,857	8,373	54,069	1,087
chr14	474,741	11,533	63,910	1,529
chr15	679,208	14,628	94,927	1,985
chr16	563,611	11,051	77,214	1,549
chr17	603,932	11,190	80,431	1,520

Table S7 List of the 148 sunflower accessions used for haplotype analysis of HaNAC7.

Haplotype	Accession	Type	Country	Provence	Plant height in 2024 (cm)	Plant height in 2025 (cm)
Hap1	Jikui1260	Confectionery	China	Jilin	204.57	196.84
Hap1	Jikui2510	Confectionery	China	Jilin	198.00	192.05
Hap1	Jikui125	Confectionery	China	Jilin	160.33	159.61
Hap1	Jikui6	Confectionery	China	Jilin	268.91	249.60
Hap1	JikuiSandaomei	Confectionery	China	Jilin	315.58	270.38
Hap1	Liaoning 2119628	Confectionery	China	Liaoning	247.59	250.45
Hap1	liaoning 2340841	Confectionery	China	Liaoning	235.88	206.60
Hap1	Elunchunquanhei	Confectionery	China	Inner Mongolia	225.59	196.80
Hap1	Neimenggu84-14	Confectionery	China	Inner Mongolia	248.99	241.03
Hap1	Bakui-120	Confectionery	China	Inner Mongolia	218.19	221.16
Hap1	76-64A	Confectionery	China	Inner Mongolia	152.01	159.73
Hap1	Bakui89-2	Confectionery	China	Inner Mongolia	228.34	234.25
Hap1	Bakui89-3	Confectionery	China	Inner Mongolia	230.37	236.80
Hap1	Lingqiubaikuihua	Confectionery	China	Shanxi	237.85	254.49
Hap1	Lingqiubaikuihua	Confectionery	China	Shanxi	255.32	251.65
Hap1	Jiaxiankuihua	Confectionery	China	Shaanxi	235.07	198.99
Hap1	2202B	Confectionery	China	Liaoning	185.44	184.98
Hap1	2230R	Confectionery	China	Liaoning	163.21	168.10
Hap1	2202B	Confectionery	China	Liaoning	197.93	207.16
Hap1	J247-1	Confectionery	China	Liaoning	205.52	175.38
Hap1	J192-2	Confectionery	China	Liaoning	226.82	193.16
Hap1	J192-4	Confectionery	China	Liaoning	209.56	204.08
Hap1	Xinjiang Pishan	Confectionery	China	Xinjiang	215.32	201.06
Hap1	Xinjiang Yanqi	Confectionery	China	Xinjiang	247.00	240.54
Hap1	8523	Oilseed	Argentina	-	214.73	228.84
Hap1	8527	Oilseed	Argentina	-	182.88	168.39
Hap1	J13-1	Oilseed	Argentina	-	221.75	188.47
Hap1	J14-1	Oilseed	Argentina	-	212.36	212.26
Hap1	Hetaohuakui2	Oilseed	China	Inner Mongolia	261.42	224.43
Hap1	Neimengguyoukui	Oilseed	China	Inner Mongolia	268.37	244.66
Hap1	Neimengguyoukui	Oilseed	China	Inner Mongolia	236.41	222.09
Hap1	J190-8	Oilseed	China	Liaoning	174.66	166.72
Hap1	J189-1	Oilseed	China	Liaoning	157.33	145.16
Hap1	J76-2	Oilseed	China	Liaoning	138.74	134.30
Hap1	J91-1	Oilseed	China	Liaoning	178.84	158.22
Hap1	Balkan-1	Oilseed	former Yugoslavia	-	209.67	219.01
Hap1	Yugoslavia 1MPIRA	Oilseed	former Yugoslavia	-	222.55	195.70
Hap1	F(-)-20	Oilseed	USA	-	235.16	203.10
Hap1	OH398	Oilseed	USA	-	227.88	193.29

Hap2	031B	Confectionery	China	Liaoning	160.06	155.87
Hap2	Dapiankuihua	Confectionery	China	Inner Mongolia	167.76	151.11
Hap2	2234R	Confectionery	China	Liaoning	141.31	150.89
Hap2	z1061	Confectionery	China	Liaoning	161.60	150.60
Hap2	J193-6	Confectionery	China	Liaoning	164.53	147.91
Hap2	J198-1	Confectionery	China	Liaoning	163.64	151.89
Hap3	Nongyuan91-11	Confectionery	China	Inner Mongolia	126.73	119.29
Hap3	Tongxiankuihua	Confectionery	China	Heilongjiang	136.80	133.49
Hap3	Xinjiang Benlei	Confectionery	China	Xinjiang	119.99	115.00
Hap3	J58-2	Oilseed	China	Liaoning	154.41	134.43
Hap3	Jinxiangyoukui	Oilseed	China	Shandong	181.44	169.76
Hap3	J186-7	Oilseed	China	Liaoning	124.85	123.55
Hap3	J181-2	Oilseed	China	Liaoning	176.93	164.55
Hap3	J66-8	Oilseed	China	Liaoning	108.57	105.94
Hap4	Hulunbeierbaimei	Confectionery	China	Inner Mongolia	163.75	157.88
Hap4	Bakui-89	Confectionery	China	Inner Mongolia	210.00	201.66
Hap4	Changlixiang	Confectionery	China	Inner Mongolia	176.32	165.31
Hap4	Zaoshubaimei	Confectionery	China	Inner Mongolia	176.53	177.86
Hap4	Nongyuan87-18	Confectionery	China	Inner Mongolia	191.43	163.68
Hap4	Abeikuihua	Confectionery	China	Sichuan	180.21	181.21
Hap4	Xinjiang Ake	Confectionery	China	Xinjiang	168.23	178.92
Hap4	8527	Oilseed	Argentina	-	175.35	182.42
Hap4	Ohara4a	Oilseed	Argentina	-	220.37	194.18
Hap4	Yugoslavia HeiKe	Oilseed	former Yugoslavia	-	175.65	165.00
Hap4	Yugoslavia Aiganxiangrikui	Oilseed	former Yugoslavia	-	174.18	154.41
Hap4	Yugoslavia DengTa	Oilseed	former Yugoslavia	-	152.84	149.44
Hap4	OLAIRSOL-1	Oilseed	Germany	-	157.45	167.12
Hap4	DO164	Oilseed	Germany	-	187.61	172.07
Hap4	OLAIRSOL-2	Oilseed	Germany	-	162.36	146.83
Hap4	Mexico P4	Oilseed	Mexico	-	156.02	149.82
Hap4	Mexico P12	Oilseed	Mexico	-	179.75	164.52
Hap4	Romania R018	Oilseed	Romania	-	212.48	206.09
Hap4	F(-)-25	Oilseed	USA	-	157.22	145.64
Hap4	F(-)-12	Oilseed	USA	-	173.35	177.47
Hap4	F(-)-22	Oilseed	USA	-	182.11	180.93
Hap5	Tai2	Confectionery	China	Heilongjiang	234.95	204.14
Hap5	Jikui81	Confectionery	China	Jilin	211.66	196.07
Hap5	Jikui121	Confectionery	China	Jilin	219.31	214.43
Hap5	MajiagangHeikui	Confectionery	China	Jilin	200.70	181.87
Hap5	Nongyuan87-5-2	Confectionery	China	Inner Mongolia	257.72	228.91
Hap5	Wuchuangkuihua	Confectionery	China	Inner Mongolia	209.83	220.94

Hap5	Heidapiankuihua	Confectionery	China	Inner Mongolia	243.05	240.03
Hap5	Bakui89-1	Confectionery	China	Inner Mongolia	210.98	197.02
Hap5	Dapiankuihua	Confectionery	China	Inner Mongolia	276.67	288.41
Hap5	Snadaomei	Confectionery	China	Shanxi	216.92	193.06
Hap5	2226R	Confectionery	China	Liaoning	191.09	187.21
Hap5	z277	Confectionery	China	Liaoning	181.52	185.10
Hap5	z1032	Confectionery	China	Liaoning	186.14	175.56
Hap5	J236-2	Confectionery	China	Liaoning	168.80	153.27
Hap5	Xinjiang Kuerle	Confectionery	China	Xinjiang	164.18	151.18
Hap5	J46-2	Oilseed	China	Liaoning	118.44	115.57
Hap5	J182-1	Oilseed	China	Liaoning	166.32	158.76
Hap5	151R	Oilseed	China	Liaoning	137.62	135.37
Hap5	France Oil-1	Oilseed	France	-	171.19	160.49
Hap5	Mexico P13	Oilseed	Mexico	-	189.47	168.97
Hap5	Romania R022	Oilseed	Romania	-	152.97	158.82
Hap6	2405R	Confectionery	China	Liaoning	203.24	179.45
Hap6	Kuihua	Confectionery	China	Heilongjiang	213.09	220.36
Hap6	Heijing12	Confectionery	China	Heilongjiang	227.09	211.63
Hap6	Qianjingheihua	Confectionery	China	Heilongjiang	242.31	235.50
Hap6	Jikui125	Confectionery	China	Jilin	215.84	209.36
Hap6	Jikui6	Confectionery	China	Jilin	213.97	194.67
Hap6	Jikui49	Confectionery	China	Jilin	239.28	228.87
Hap6	Jikui1341	Confectionery	China	Jilin	167.24	167.49
Hap6	Xiaoheisandaomen	Confectionery	China	Inner Mongolia	229.69	238.00
Hap6	Nongyuan87-18	Confectionery	China	Inner Mongolia	202.80	187.49
Hap6	Nongyuan87-5-1	Confectionery	China	Inner Mongolia	225.52	206.00
Hap6	Sandaobai	Confectionery	China	Inner Mongolia	244.74	259.25
Hap6	Changlixiang	Confectionery	China	Inner Mongolia	226.81	202.67
Hap6	Nongyuan87-18	Confectionery	China	Inner Mongolia	201.25	204.40
Hap6	Hetaohuamei	Confectionery	China	Inner Mongolia	252.10	226.17
Hap6	Sandaomei	Confectionery	China	Inner Mongolia	257.39	225.89
Hap6	Daheiguazi	Confectionery	China	Inner Mongolia	283.08	263.28
Hap6	Sandaobai	Confectionery	China	Inner Mongolia	303.88	321.25
Hap6	Sandaomei	Confectionery	China	Shanxi	222.19	226.11
Hap6	Sandaomei	Confectionery	China	Shanxi	215.84	211.04
Hap6	Lingqiubaikuihua	Confectionery	China	Shanxi	235.23	237.48
Hap6	Lingqiubaikuihua	Confectionery	China	Shanxi	253.78	251.63
Hap6	z681	Confectionery	China	Liaoning	228.54	199.77
Hap6	2233R	Confectionery	China	Liaoning	199.30	171.78
Hap6	2216B	Confectionery	China	Liaoning	176.04	152.65
Hap6	2220B	Confectionery	China	Liaoning	198.77	195.13
Hap6	z221	Confectionery	China	Liaoning	164.39	173.44
Hap6	z260	Confectionery	China	Liaoning	161.73	156.56
Hap6	z280	Confectionery	China	Liaoning	214.19	184.25

Hap6	z986	Confectionery	China	Liaoning	203.21	171.67
Hap6	z1013	Confectionery	China	Liaoning	173.70	167.81
Hap6	z1018	Confectionery	China	Liaoning	203.11	175.77
Hap6	z1050	Confectionery	China	Liaoning	194.77	183.33
Hap6	z1074	Confectionery	China	Liaoning	162.99	158.41
Hap6	z1056	Confectionery	China	Liaoning	177.81	166.37
Hap6	J199-1	Confectionery	China	Liaoning	169.21	181.41
Hap6	Chongqin Taihe	Confectionery	China	Chongqing	145.33	145.84
Hap6	J12-4	Oilseed	Argentina	-	208.05	186.28
Hap6	J16-4	Oilseed	Argentina	-	220.59	199.10
Hap6	Canada 85500	Oilseed	Canada	-	199.40	190.72
Hap6	Canada 118706	Oilseed	Canada	-	167.45	172.81
Hap6	J60-1	Oilseed	China	Liaoning	143.38	132.55
Hap6	412B	Oilseed	China	Liaoning	256.79	235.98
Hap6	F60R	Oilseed	China	Liaoning	190.88	195.03
Hap6	Hetaohuakui1	Oilseed	China	Inner Mongolia	249.00	238.64
Hap6	J92-5	Oilseed	China	Liaoning	176.24	171.29
Hap6	C6	Oilseed	China	Liaoning	161.68	169.56
Hap6	L405R	Oilseed	China	Liaoning	158.27	146.03
Hap6	Yugoslavia S-25	Oilseed	former Yugoslavia	-	196.42	189.76
Hap6	France Oil-2	Oilseed	France	-	177.98	163.55
Hap6	Fu-1	Oilseed	France	-	208.74	200.86
Hap6	Mexico P	Oilseed	Mexico	-	205.82	194.13
Hap6	Romania R055	Oilseed	Romania	-	180.53	174.06

Table S8 Allelic states, functional annotations, and haplotype assignments of the four *HaNAC7* polymorphic sites in the parental lines 150A and PT326.

SNP position	Ref/ Alt	150A allele	PT326 allele	Functional annotation	150A-associated haplotype	PT326-associated haplotype
chr15:107,284,400	G/A	G	A	Downstream gene variant	Hap6	Hap1
chr15:107,285,211	T/C	T	C	Missense variant		
chr15:107,288,600	C/T	T	C	Upstream gene variant		
chr15:107,289,520	A/G	G	A	Upstream gene variant		

Table S9 Single-marker association analysis of the four *HaNAC7* polymorphic sites with plant height in 148 sunflower accessions in 2024 and 2025.

SNP	Allele	Plant height in 2024				Plant height in 2025			
		Mean (cm)	SD (cm)	t value	*P* value	Mean (cm)	SD (cm)	t value	*P* value
chr15:107,289,520	A	195.83	40.01	-0.935	0.351	186.10	37.45	-1.042	0.299
	G	201.86	35.87			192.56	36.01		
chr15:107,288,600	C	193.55	39.76	-2.004	0.047	183.91	37.22	-2.129	0.035
	T	206.62	34.63			197.22	35.05		
chr15:107,285,211	C	199.22	36.96	0.279	0.781	189.25	33.68	0.168	0.867
	T	197.44	39.73			188.22	39.49		
chr15:107,284,400	A	198.96	44.77	0.171	0.864	188.19	41.42	-0.12	0.904
	G	197.83	34.59			188.95	34.35		
